# Author Correction: Quantum clocks and the temporal localisability of events in the presence of gravitating quantum systems

**DOI:** 10.1038/s41467-020-20105-3

**Published:** 2020-11-25

**Authors:** Esteban Castro-Ruiz, Flaminia Giacomini, Alessio Belenchia, Časlav Brukner

**Affiliations:** 1 Faculty of Physics, Vienna Center for Quantum Science and Technology (VCQ), University of Vienna, Boltzmanngasse 5, A-1090 Vienna, Austria; 2 Institute for Quantum Optics and Quantum Information (IQOQI), Austrian Academy of Sciences, Boltzmanngasse 3, A-1090 Vienna, Austria; 3 Centre for Quantum Information and Communication, École polytechnique de Bruxelles, Université libre de Bruxelles, CP 165, 1050 Brussels, Belgium; 4 Perimeter Institute for Theoretical Physics, 31 Caroline St. N, Waterloo, ON N2L 2Y5 Canada; 5 Centre for Theoretical Atomic, Molecular, and Optical Physics, School of Mathematics and Physics, Queen’s University, Belfast, BT7 1NN UK

**Keywords:** Quantum information, Theoretical physics

Correction to: *Nature Communications* 10.1038/s41467-020-16013-1, published online 29 May 2020.

The original version of this Article contained an error in the fifth sentence of the abstract, which was previously incorrectly given as ‘This relativity ia a signature of an indefinite metric, where events can occur in an indefinite causal order’. The correct version states ‘This relativity is a signature of an indefinite metric, where events can occur in an indefinite causal order’. This has been corrected in both the PDF and HTML versions of the Article.

