# Supplementary material for: Quantum clocks and the temporal localisability of events in the presence of gravitating quantum systems
Source: Nat Commun. 2020 May 29;11:2672. doi: 10.1038/s41467-020-16013-1 (PMC7260228; doi:10.1038/s41467-020-16013-1)
Supplement: Supplementary file 1 — Supplementary Information [file 41467_2020_16013_MOESM1_ESM.pdf]

Quantum clocks and the temporal localisability of events in the presence of gravitating quantum systems: Supplementary notes

Castro-Ruiz et al.

SUPPLEMENTARY NOTE 1: TROTTER FORMULAS

The history state satisfies the constraint equation

$$\hat{C}\,|\Psi\rangle = 0, \tag{1}$$

which we can solve by group averaging

$$|\Psi\rangle = \int \mathrm{d}\alpha\, e^{-i\alpha\hat{C}}\,|\varphi\rangle\,. \tag{2}$$

In order to find solutions to Eq. (1) it is useful to derive Trotter formulas for the exponent in Eq. (2). In this Supplementary Note we derive the Trotter formulas used to find the history states in the main text.

Basic formula

Consider a Hilbert space $\mathcal{H}$ with a tensor factorisation of the form $\mathcal{H} = \mathcal{H}_\mathrm{C} \otimes \mathcal{H}_\mathrm{R}$. Let $\hat{T}$ and $\hat{H}$ be operators on $\mathcal{H}_\mathrm{C}$ satisfying $[\hat{T}, \hat{H}] = i$, and let $\hat{f}(s)$ be an operator-valued function of the parameter s acting on $\mathcal{H}_\mathrm{R}$. If we replace the argument in $\hat{f}(s)$ by the operator $\hat{T}$, we obtain an operator valued function, $\hat{f}(\hat{T})$, of the operator $\hat{T}$ acting on $\mathcal{H}$. In this setting, the basic Trotter formula reads

$$e^{-i\alpha\left(\hat{H}+\hat{f}(\hat{T})\right)} = e^{-i\alpha\hat{H}}\mathrm{T}e^{-i\int_0^\alpha \mathrm{d}s\,\hat{f}(s+\hat{T})}, \tag{3}$$

where T denotes the time-ordering operator, which acts as $\mathrm{T}\hat{f}(s_1)\hat{f}(s_2) = \Theta(s_2-s_1)\hat{f}(s_2)\hat{f}(s_1) + \Theta(s_1-s_2)\hat{f}(s_1)\hat{f}(s_2)$ for any operator-valued function $\hat{f}$ of s.

In order to prove Eq. (3), we use Trotter formula,

$$e^{-i\alpha\left(\hat{H}+\hat{f}(\hat{T})\right)} = \lim_{N\to\infty}\left(e^{-i\frac{\alpha}{N}\hat{H}}e^{-i\frac{\alpha}{N}\hat{f}(\hat{T})}\right)^N, \tag{4}$$

applied to the generic basis vector $|t,r\rangle \in \mathcal{H}$, where $\hat{T}\,|t\rangle = t\,|t\rangle$ and $|r\rangle \in \mathcal{H}_\mathrm{R}$. After successive application of $L = e^{-i\frac{\alpha}{N}\hat{H}}e^{-i\frac{\alpha}{N}\hat{f}(\hat{T})}$ to $|t,r\rangle$, one finds

$$L^N\,|t,r\rangle = \; e^{-i\alpha\hat{H}}\overleftarrow{\Pi}_{k=1}^{N} e^{-i\frac{\alpha}{N}\hat{f}\left(\hat{T}+(k-1)\frac{\alpha}{N}\right)}\,|t,r\rangle\,. \tag{5}$$

Note that the right hand side (rhs) of Eq. (5) can be seen as a ($\overleftarrow{\Pi}$-ordered) Riemann sum, which, in the limit $N \longrightarrow \infty$, gives

$$\lim_{N\to\infty} L^N\,|t,r\rangle = e^{-i\alpha\hat{H}}\mathrm{T}e^{-i\int_t^{t+\alpha}\mathrm{d}s\,\hat{f}(s)}\,|t,r\rangle\,. \tag{6}$$

The change of integration variable $s \longrightarrow s-t$ gives Eq. (3) applied to the basis vector $|t,r\rangle$. At this point, we can substitute the parameter t for the operator $\hat{T}$. By linearity, Eq. (3) holds.

Variation 1: gravitating quantum clocks

Consider a Hilbert space of the form $\mathcal{H} = \mathcal{H}_\mathrm{A} \otimes \mathcal{H}_\mathrm{B} \otimes \mathcal{H}_\mathrm{R}$, with operators $\hat{T}_\mathrm{I}$ and $\hat{H}_\mathrm{I}$ on $\mathcal{H}_\mathrm{I}$ satisfying $[\hat{T}_\mathrm{I}, \hat{H}_\mathrm{I}] = i$, for $\mathrm{I} = \mathrm{A},\ \mathrm{B}$, and an operator-valued function $\hat{f}(\hat{T}_\mathrm{A})$, where $\hat{f}(s)$ is an operator on $\mathcal{H}_\mathrm{R}$ for all s. A variation of Eq. (3), relevant to the case considered in Events for gravitating quantum clocks, is

$$e^{-i\alpha\left(\hat{H}_\mathrm{A}+\hat{H}_\mathrm{B}+\lambda\hat{H}_\mathrm{A}\hat{H}_\mathrm{B}+(1+\lambda\hat{H}_\mathrm{B})\hat{f}(\hat{T}_\mathrm{A})\right)} = e^{-i\alpha(\hat{H}_\mathrm{A}+\hat{H}_\mathrm{B}+\lambda\hat{H}_\mathrm{A}\hat{H}_\mathrm{B})}\mathrm{T}e^{-i\int_0^\alpha \mathrm{d}s(1+\lambda\hat{H}_\mathrm{B})\,\hat{f}(s(1+\lambda\hat{H}_\mathrm{B})+\hat{T})}. \tag{7}$$

In order to prove this formula, we proceed by dividing the $[0,\alpha]$ interval into N equal parts, as in the previous case, and applying successively the operator $L = e^{-i\frac{\alpha}{N}(\hat{H}_\mathrm{A}+\hat{H}_\mathrm{B}+\lambda\hat{H}_\mathrm{A}\hat{H}_\mathrm{B})}e^{-i\frac{\alpha}{N}\hat{f}(\hat{T}_\mathrm{A})(1+\lambda\hat{H}_\mathrm{B})}$ to the basis vector $|t,\omega,r\rangle$, with $\hat{T}_\mathrm{A}\,|t\rangle = t\,|t\rangle$, $\hat{H}_\mathrm{B}\,|\omega\rangle = \omega\,|\omega\rangle$ and $|r\rangle \in \mathcal{H}_\mathrm{R}$. Following the same steps as in the previous case, we obtain, in the limit $N \longrightarrow \infty$,

$$\lim_{N\to\infty} L^N\,|t,\omega,r\rangle = e^{-i\alpha(\hat{H}_\mathrm{A}+\hat{H}_\mathrm{B}+\lambda\hat{H}_\mathrm{A}\hat{H}_\mathrm{B})}\mathrm{T}e^{-i\int_t^{t+\alpha(1+\lambda\omega)}\mathrm{d}s\,\hat{f}(s)}\,|t,\omega,r\rangle\,. \tag{8}$$

Eq. (7) results after the change of integration variable $s \longrightarrow (s-t)/(1+\lambda\omega)$ and the substitution of the parameters t and ω for the operators $\hat{T}_\mathrm{A}$ and $\hat{H}_\mathrm{B}$.

Variation 2: gravitational quantum switch

In this case, the Hilbert space is $\mathcal{H} = \mathcal{H}_{\rm A} \otimes \mathcal{H}_{\rm B} \otimes \mathcal{H}_{\rm C} \otimes \mathcal{H}_M \otimes \mathcal{H}_{\rm R}$, with operators $\hat{T}_{\rm I}$ and $\hat{H}_{\rm I}$ on $\mathcal{H}_{\rm I}$ satisfying $[\hat{T}_{\rm I}, \hat{H}_{\rm I}] = i$, for I = A, B, C and operator-valued functions $\hat{f}_{\rm I}(\hat{T}_{\rm I})$, where $\hat{f}_{\rm I}(s)$ an operator on $\mathcal{H}_{\rm R}$ for all s and for I = A, B. Consider, in addition, operators $\hat{\phi}_{\rm I}$ on $\mathcal{H}_M$, with mutual eigenvectors $|{\rm Q}\rangle \in \mathcal{H}_{\rm R}$ satisfying $\hat{\phi}_{\rm I} |{\rm Q}\rangle = \phi_{\rm I}^{\rm Q} |{\rm Q}\rangle$ for I = A, B, C. The relevant formula for the case of the gravitational quantum switch is

$$e^{-i\alpha\left(\sum_{\rm I=A,B,C} \hat{H}_{\rm I}(1+\hat{\phi}_{\rm I})+\sum_{\rm I=A,B} \hat{f}_{\rm I}(\hat{T}_{\rm I})(1+\hat{\phi}_{\rm I})\right)} = e^{-i\sum_{\rm I=A,B,C} \hat{H}_{\rm I}(1+\hat{\phi}_{\rm I})} \mathrm{T} e^{-i\sum_{\rm I=A,B} \int_0^\alpha {\rm d}s (1+\hat{\phi}_{\rm I})\, \hat{f}(s(1+\hat{\phi}_{\rm I})+\hat{T}_{\rm I})}. \tag{9}$$

We can prove Eq. (9) by following the same steps as in the two previous formulas. This time, we apply successively the operator $L = e^{-i\frac{\alpha}{N}\sum_{\rm I=A,B,C} \hat{H}_{\rm I}(1+\hat{\phi}_{\rm I})} e^{-i\frac{\alpha}{N}\sum_{\rm I=A,B} \hat{f}_{\rm I}(\hat{T}_{\rm I})(1+\hat{\phi}_{\rm I})}$ to the basis vector $|t_{\rm A}, t_{\rm B}, t_{\rm C}, {\rm Q}, {\rm R}\rangle$, where $\hat{T}_{\rm I} |t_{\rm I}\rangle = t_{\rm I} |t_{\rm I}\rangle$ for I = A, B, C.

SUPPLEMENTARY NOTE 2: DERIVATION OF HISTORY STATES WITH RESPECT TO DIFFERENT TIME REFERENCE FRAMES

In this Supplementary Note we will show in detail how to derive the history states used in Non interacting clocks, Events with respect to gravitationally interacting clocks, and Methods (Gravitational quantum switch) in the main text. We will proceed by using Eq. (2) in order to obtain the "perspective neutral" representation of $|\Psi\rangle$ and then changing coordinates to a specific time reference frame.

History state for non interactiong clocks

We begin by analysing the simplest case, that is, the one discussed in Non interacting clocks. The constraint is

$$\hat{\rm C} = \hat{H}_{\rm A} + \hat{H}_{\rm B} + \hat{f}_{\rm A}(\hat{T}_{\rm A}) + \hat{f}_{\rm A}(\hat{T}_{\rm A}) \tag{10}$$

First we obtain the history state $|\Psi\rangle$, satisfying $\hat{\rm C}|\Psi\rangle = 0$, by using the results of Supplementary Note 1. By slightly generalising Eq. (3), we obtain:

$$|\Psi\rangle = \int {\rm d}\alpha\, e^{-i\alpha(\hat{H}_{\rm A}+\hat{H}_{\rm B})} \mathrm{T}\, e^{-i\int_0^\alpha {\rm d}s\, \left(\hat{f}_{\rm A}(s+\hat{T}_{\rm A})+\hat{f}_{\rm B}(s+\hat{T}_{\rm B})\right)} |\varphi\rangle\,. \tag{11}$$

Let us now write $|\varphi\rangle$ explicitly as

$$|\varphi\rangle = \int {\rm d}t'_{\rm A} {\rm d}t'_{\rm B} \varphi(t'_{\rm A}, t'_{\rm B})\, |t'_{\rm A}, t'_{\rm B}\rangle_{\rm AB} \otimes |\chi\rangle_{\rm R} \tag{12}$$

and insert Eq. (12) into Eq. (11). Next, we define the variable $t_{\rm A}(\alpha) = t'_{\rm A} + \alpha$ and change variables to eliminate α in favour of $t_{\rm A}$. Then, the history state reads

$$|\Psi\rangle = \int {\rm d}t_{\rm A} {\rm d}t'_{\rm A} {\rm d}t'_{\rm B}\, \varphi(t'_{\rm A}, t'_{\rm B})\, \mathrm{T}\, e^{-i\int_0^{t_{\rm A}-t'_{\rm A}} {\rm d}s\, (\hat{f}_{\rm A}(s+t'_{\rm A})+\hat{f}_{\rm B}(s+t'_{\rm B}))} |t_{\rm A}\rangle_{\rm A} \otimes |t_{\rm A} + t'_{\rm B} - t'_{\rm A}\rangle_{\rm B} \otimes |\chi\rangle_{\rm R}\,. \tag{13}$$

Now, in the integral in s, we make the change of variable $s \longrightarrow s - t'_{\rm A}$. We next divide the resulting integral in the exponent into two integrals, one from 0 to $t_{\rm A}$ and another one from $t'_{\rm A}$ to 0. After arranging terms, turning the necessary c-numbers into operators, and using the fact that the time ordering operator T allows us to commute terms acted upon by it, we obtain

$$|\Psi\rangle = \int {\rm d}t_{\rm A}\; |t_{\rm A}\rangle_{\rm A} \otimes e^{-it_{\rm A}\hat{H}_{\rm B}}\, \mathrm{T}\, e^{-i\int_0^{t_{\rm A}} {\rm d}s\, (\hat{f}_{\rm A}(s)+\hat{f}_{\rm B}(s+\hat{T}_{\rm B}))} |\psi_{\rm A}(0)\rangle_{\bar{\rm A}}, \tag{14}$$

where

$$|\psi_{\rm A}(0)\rangle_{\bar{\rm A}} = \int {\rm d}t'_{\rm A} {\rm d}t'_{\rm B}\, \mathrm{T}\, e^{i\int_0^{t'_{\rm A}} {\rm d}s (\hat{f}_{\rm A}(s)+\hat{f}_{\rm B}(s+\hat{T}_{\rm B}))} \varphi(t'_{\rm A}, t'_{\rm B})\; |t'_{\rm B} - t'_{\rm A}\rangle_{\rm B} \otimes |\chi\rangle_{\rm R}\,. \tag{15}$$

Eq. (14) is precisely the history state with respect to A presented in Non interacting clocks in the main text. Clearly, following the same steps, we can compute the history state and the initial state from the point of view of B. Moreover,

by symmetry, it is easy to check that $|\psi_{\rm B}(0)\rangle_{\bar{\rm B}}$ can be obtained from Eq. (15) by swapping the labels A and B everywhere but in the argument of φ. Now, as mentioned in Non interacting clocks, it is physically meaningful to assume that the initial state of clock B, from the point of view of A, as well as the initial state of clock A, from the point of view of B, are both centred around times that are strictly smaller than the times $t^*_{\rm I}$, at which S and the ancillas interact. Both conditions can be achieved by demanding that $\varphi(t'_{\rm A}, t'_{\rm B})$ be supported in a region $D_\varphi^{\times 2}$, where $D_\varphi \subsetneq [-\epsilon, \min_{\rm I=A,B}\{t^*_{\rm I}\}]$, with $\epsilon > 0$. Moreover, the state $|\psi_{\rm A}(0)\rangle_{\bar{\rm A}}$ is normalised if and only if $|\psi_{\rm B}(0)\rangle_{\bar{\rm B}}$ is normalised as well. In the case where $\varphi(t'_{\rm A}, t'_{\rm B}) = \delta(t'_{\rm A})\varphi(t'_{\rm B})$, where $\varphi(t'_{\rm B})$ is a symmetric function, centred around $t'_{\rm B} = 0$ and supported on D_φ, $|\psi_{\rm A}(0)\rangle_{\bar{\rm A}}$ and $|\psi_{\rm B}(0)\rangle_{\bar{\rm B}}$ have exactly the same form, as can be seen by inserting this choice of φ into Eq. (15) and using the symmetry of $\varphi(t'_{\rm B})$ together with the fact that the initial states of A and B are related by a change of labels. This is the choice of initial state that we used for our discussion in Non interacting clocks in the main text.

History state for gravitating quantum clocks

We will now derive explicitly the history state in the time reference frame of A presented in Events with respect to gravitationally interacting clocks. The history state in the time reference frame of C is easily obtained following the same logic. Consider the constraint

$$\hat{C} = \hat{H}_{\rm A} + \hat{H}_{\rm B} + \hat{H}_{\rm C} + \lambda \hat{H}_{\rm A}\hat{H}_{\rm B} + \hat{f}(\hat{T}_{\rm A})(1 + \lambda \hat{H}_{\rm B}). \tag{16}$$

By using Eq. (7) and writing down explicitly

$$|\varphi\rangle = \int {\rm d}t'_{\rm A}{\rm d}\omega_{\rm B}{\rm d}t'_{\rm C}\varphi(t'_{\rm A}, \omega_{\rm B}, {\rm d}t'_{\rm C})\, |t'_{\rm A}, \omega_{\rm B}, t'_{\rm C}\rangle_{\rm ABC} \otimes |\chi\rangle_{\rm R} \tag{17}$$

in Eq. (2), we obtain, after some manipulation

$$|\Psi\rangle = \int {\rm d}\alpha {\rm d}t'_{\rm A}{\rm d}\omega_{\rm B}{\rm d}t'_{\rm C}\, \varphi(t'_{\rm A}, \omega_{\rm B}, {\rm d}t'_{\rm C}) e^{-i\alpha\omega_{\rm B}}\, {\rm T}\, e^{-i\int_0^\alpha {\rm d}s(1+\lambda\omega_{\rm B})\hat{f}(s(1+\lambda\omega_{\rm B})+t'_{\rm A})}\, |t'_{\rm A} + \alpha(1+\lambda\omega_{\rm B}), \omega_{\rm B}, t'_{\rm C} + \alpha\rangle_{\rm ABC} \otimes |\chi\rangle_{\rm R}\,. \tag{18}$$

We now define the new variable $\tau_{\rm A}(\alpha) := t'_{\rm A} + \alpha(1+\lambda\omega_{\rm B})$ and change variables to eliminate α in favour of $\tau_{\rm A}$. Note that, by doing this, we change the integration measure concerning the variable $\omega_{\rm B}$, which now reads ${\rm d}\mu(\omega_{\rm B}) = {\rm d}\omega_{\rm B}/|1+\lambda\omega_{\rm B}|$. We assume that $\varphi(t'_{\rm A}, \omega_{\rm B}, {\rm d}t'_{\rm C})$ is such that no divergencies in the new integration measure occur. Following the same steps leading to Eq. (14), we also do the change of integration variable $s \longrightarrow (s - t'_{\rm A})/(1 + \lambda\omega_{\rm B})$. After some manipulations analogous to those used in the non interacting case, we arrive at

$$|\Psi\rangle = \int {\rm d}\tau_{\rm A}\; |\tau_{\rm A}\rangle \otimes {\rm T}\, e^{-i\int_0^{\tau_{\rm A}} {\rm d}s\left(\frac{\hat{H}_{\rm B}+\hat{H}_{\rm C}}{1+\lambda\hat{H}_{\rm B}} + \hat{f}(s)\right)}\, |\psi_{\rm A}(0)\rangle_{\bar{\rm A}}, \tag{19}$$

where the initial state is given by

$$|\psi_{\rm A}(0)\rangle_{\bar{\rm A}} = \int {\rm d}t'_{\rm A}{\rm d}\mu(\omega_{\rm B}){\rm d}t'_{\rm C}\varphi(t'_{\rm A}, \omega_{\rm B}, t'_{\rm C}) e^{i\int_0^{t_{\rm A}} {\rm d}s\, \frac{\hat{H}_{\rm B}+\hat{H}_{\rm C}}{1+\lambda\hat{H}_{\rm B}} + \hat{f}(s)}\, |\omega_{\rm B}, t'_{\rm C}\rangle_{\rm BC} \otimes |\chi\rangle_{\rm R}\,. \tag{20}$$

Eq. (19) is precisely the history state, in the time reference frame of A, presented in Events with respect to gravitationally interacting clocks in the main text.

The history state in the time reference frame of C is obtained following the same steps leading to Eq. (19) but with the changes of variables $\tau_{\rm C}(\alpha) := t'_{\rm C} + \alpha$ and $s \longrightarrow s - t'_{\rm C}$. The initial state is calculated in exactly the same way as that in Eq. (20).

History state for the gravitational quantum switch

Let us now show how to derive the history state, from the perspective of clock A, presented in Methods (Gravitational quantum switch). The history state from the perspective of clock C is obtained in a completely analogous way. Consider the constraint

$$\hat{C} = \sum_{\rm I} \hat{H}_{\rm I}(1 + \hat{\Phi}_{\rm I}) + \sum_{\rm I} \hat{f}_{\rm I}(\hat{T}_{\rm I})(1 + \hat{\Phi}_{\rm I}). \tag{21}$$

For the state

$$|\varphi\rangle = \sum_{\rm Q} \int {\rm d}t'_{\rm A}{\rm d}t'_{\rm B}{\rm d}t'_{\rm C}\, \varphi_{\rm Q}(t'_{\rm A}, t'_{\rm B}, t'_{\rm C})\, |t'_{\rm A}, t'_{\rm B}, t'_{\rm C}\rangle_{\rm ABC} \otimes |{\rm Q}\rangle_{\rm M} \otimes |\chi\rangle_{\rm Sab}, \tag{22}$$

Eqs. (2) and (9) lead to

$$|\Psi\rangle = \sum_{\rm Q} \int \Pi_{\rm I}{\rm d}t_{\rm I}\varphi_{\rm Q}(\{t'_{\rm I}\})\, {\rm T}\, e^{-i\sum_{\rm I=A,B}\int_0^\alpha {\rm d}s(1+\Phi^{\rm Q}_{\rm I})f(s(1+\hat{\Phi}^{\rm Q}_{\rm I})+t'_{\rm I})} \bigotimes_{\rm I=A,B,C} \left|t'_{\rm I} + \alpha(1+\Phi^{\rm Q}_{\rm I})\right\rangle_{\rm I} \otimes |{\rm Q},\chi\rangle_{\rm MSab}\,. \tag{23}$$

Here, we have used the simplifed notation $\{t'_{\rm I}\}$ to refer to all three time variables $t'_{\rm A}$, $t'_{\rm B}$ and $t'_{\rm C}$. Following the previous two cases, we now define the variable $\tau_{\rm A}(\alpha) := t'_{\rm A} + \alpha(1+\Phi^{\rm Q}_{\rm A})$, in order to eliminate the variable α in favour of $\tau_{\rm A}$. This will enforce a change in the integration measure —we have to include now a factor of $1/|1+\Phi^{\rm Q}_{\rm A}|$ when integrating (or summing, in this case) over the variable Q. We assume that the states $\varphi_{\rm Q}(\{t'_{\rm I}\})$ are such that no divergences occur in the integration measure. We also make the change of variables $s \longrightarrow (s-t'_{\rm A})/(1+\Phi^{\rm Q}_{\rm A})$. After some manipulations similar to those of previous cases, we get

$$|\Psi\rangle = \int {\rm d}\tau_{\rm A}\; |\tau_{\rm A}\rangle_{\rm A} \otimes {\rm T}\, e^{-i\int_0^{\tau_{\rm A}} {\rm d}s\left(\hat{f}_{\rm A}(s)+\sum_{\rm I}\hat{\Delta}(I,A)\left(\hat{H}_{\rm I}+\hat{f}_{\rm I}(s\hat{\Delta}({\rm I,A})+\hat{T}_{\rm I})\right)\right)}\, |\psi_{\rm A}(0)\rangle_{\bar{\rm A}}, \tag{24}$$

where

$$|\psi_{\rm A}(0)\rangle_{\bar{\rm A}} = \sum_{\rm Q}\int \frac{\Pi_{\rm I}{\rm d}t_{\rm I}}{|1+\Phi^{\rm Q}_{\rm A}|}\, \varphi_{\rm Q}(\{t'_{\rm I}\})\, {\rm T}\, e^{i\int_0^{\tau_{\rm A}} {\rm d}s\left(\hat{f}_{\rm A}(s)+\hat{f}_{\rm B}(s\hat{\Delta}(B,A)+\hat{T}_{\rm B})\right)} e^{i\sum_{\rm I=B,C} t'_{\rm A}\hat{\Delta}(I,A)\hat{H}_{\rm I}} \bigotimes_{\rm I=B,C} |t'_{\rm I}\rangle_{\rm I} \otimes |{\rm Q},\chi\rangle_{\rm MSab}\,. \tag{25}$$

Eq. (25) is precisely the history state for the quantum switch from Methods (Gravitational quantum switch) in the main text.

The history state from the perspective of C and the corresponding initial state are obtained in an analogous way, by defining the new variable $\tau_C(\alpha) := t'_{\rm C} + \alpha(1+\Phi^{\rm Q}_{\rm C})$ and eliminating α in favour of $\tau_{\rm C}$, and by making the change of integration variable $s \longrightarrow (s-t'_{\rm C})/(1+\Phi^{\rm Q}_{\rm C})$.

Note that in Eq. (25) as well as in the corresponding expression from the point of view of C, the order in which the operations are applied, in A's or C's reference frame, is controlled only by the time parameter corresponding to A's or C's clock. Therefore, in this approximation, A's operation will be in the causal past of B's operation if the time when A's operation is applied (in the frame of A or C) is smaller that the time when B's operation is applied. A more complete approach would have to take into account not only the times but also the light cones at each event. Then, the commutation relations between the operations applied should depend on whether the events are time-like of space-like separated. In principle, such a situation can be modelled with a family of time-dependent operators $\hat{K}^{\rm I}_{\rm iS}(\hat{T}_{\rm I})$, for the clocks I = A, B and for the ancillas i = a, b. These operators would commute if the corresponding events are outside of each other's light cones (defined with respect to each position of the mass) and not commute otherwise.
